# A cohort analysis of neonatal hospital mortality rate and predictors of neonatal mortality in a sub-urban hospital of Cameroon

**DOI:** 10.1186/s13052-017-0369-5

**Published:** 2017-06-05

**Authors:** Paul Koki Ndombo, Quinta Mua Ekei, Joel Noutakdie Tochie, Mazou Ngou Temgoua, Francky Teddy Endomba Angong, Ferdinand Ndom Ntock, Lawrence Mbuagbaw

**Affiliations:** 1 Mother and Child Center, Chantal Biya Foundation, Yaounde, Cameroon; 2 Department of Paediatrics, Faculty of Medicine and Biomedical Sciences, University of Yaounde 1, Yaounde, Cameroon; 3 Faculty of Health Sciences, University of Buea, Buea, Cameroon; 4 Faculty of Medicine and Biomedical Sciences, University of Yaounde 1, Yaounde, Cameroon; 5 Health and Human development (2HD) Research Group, Douala, Littoral Region, Cameroon; 6 Department of Pediatrics, Buea Regional Hospital, Buea, Cameroon; 7 Department of Pediatrics, Faculty of Health Sciences, University of Buea, Buea, Cameroon

**Keywords:** Newborns, Hospital neonatal mortality, Predictor, Suburban, Cameroon

## Abstract

**Background:**

In Cameroon, sustainable effort needs to be done to reduce the current neonatal mortality rate from 21 deaths per 1000 live births to the global target of fewer than ten deaths per 1000 live births by 2035. We aimed to determine the neonatal hospital mortality rate and predictors of neonatal hospital mortality (NHM) in a major referral sub-urban hospital of Cameroon in a bit to formulate interventions to curb this burden.

**Methods:**

This was a prospective cohort study consecutively enrolling all neonates admitted into the neonatology unit of the Bamenda Regional Hospital (BRH) from November 2015 to February 2016. Through interviewed questionnaires to parents and physical examination of neonates, we studied socio-demographic characteristics, antenatal history, intrapartum history and clinical findings of neonates. Neonates further underwent relevant laboratory investigations for diagnosis. All neonates were followed up till 28 days after the post-menstrual term for the neonatal outcomes. Multiple logistic regression was used to determine predictors of NHM.

**Results:**

We enrolled 332 out of 337 neonates admitted to the neonatology unit of BRH during the study period. Fifty-three percent (53%) were males. Their mean gestational age and birth weight were 36.9 ± 3.9 weeks and 2677.2 ± 923 g, respectively. The main causes of neonatal admissions were complications of preterm birth (32.2%), neonatal infections (31.3%), and birth asphyxia (14.5%). The neonatal hospital mortality rate was 15.7%. NHM was related to complications of preterm birth (69%), birth asphyxia (23%) and neonatal infections (6%). A five-minute Apgar score less than seven was the only predictor of NHM (aOR: 16.41; CI 95%: 6.35–42.47; *p* < 0.01).

**Conclusion:**

Neonatal mortality still remains a significant health problem in sub-urban Cameroon, mainly as a result of three pathologies; complications of preterm birth, birth asphyxia, and infections. There is an urgent need to revamp the current health policies through the improvement of antenatal care, skilled birth attendants, neonatal resuscitation, timely detection and treatment of complications of preterm births, birth asphyxia, and infections.

## Background

The neonatal period (the first 28 days of life) is a very delicate phase in life due to the risk of acquiring potential life-threatening diseases, and the complexity of the adaptive process of the newborn [1–3]. Global estimates show that 2.8 million neonatal deaths occurred in 2013, accounting for 44% of deaths in under-fives [4]. There is evidence suggesting a relatively rapid decline in the global under-five mortality compared to the global neonatal mortality (annual rates of reduction 4.9% vs. 2.9%) between the year 2000 and 2013 [4]. With an average neonatal mortality rate (NMR) of 41 per 1000 live births, this burden still remains unresolved in sub-Saharan Africa [5]. This is in contrast with developed countries where there is averagely 4 neonatal deaths per 1000 live births [5]. Consequently, the Every Newborn Action Plan calls for a worldwide reduction in NMR to fewer than ten deaths per 1000 live births by 2035 [6]. The way forward entails designing robust sustainable interventions to prevent and adequately treat complications of preterm birth, birth asphyxia and neonatal sepsis, which jointly account for three-quarter of neonatal deaths globally [4].

Similarly, with 21 neonatal deaths per 1000 live births recorded in Cameroon in 2015, neonatal mortality remains a considerable concern [7]. Previous studies from urban Cameroon cited infections, complications of preterm birth, birth asphyxia, and congenital malformations as the main causes of neonatal hospital mortality [8–10]. Though high birth rates occur in sub-urban Cameroon where access to neonatal health services is inadequate [11, 12], epidemiological data on neonatal mortality in this area remains scarce. Furthermore, the patterns of neonatal mortality are useful indicators of the quality of obstetrical and neonatal care in a particular setting, and their assessment ensures the estimation of the quality of health care [13, 14]. Therefore, we proposed this study to determine the neonatal hospital mortality rate and its predictors in a sub-urban setting of Cameroon. This should inform public health authorities on the current burden of neonatal mortality in order to tailor control interventions to curb this burden.

## Methods

### Study area and setting

This was a hospital-based prospective cohort study carried out from November 11th, 2015 to February 29th, 2016 at the neonatology unit of the Bamenda Regional Hospital (BRH), a secondary level health facility in Cameroon. This hospital situated in the North-West Region of Cameroon serves as a reference health facility for mother and child health care. Its average annual neonatal admission is 900 neonates. The neonatology unit is divided into three sections; a nursery for preterm and low birth weight newborns, a ward for term neonates and a ward for infants aged between two and 3 months. Only neonates admitted to the wards for preterm and low birth weight neonates, and term neonates participated in this study. Infrastructure available in this neonatology unit includes 13 incubators, 45 cots, five phototherapy lamps, two oxygen cylinders and one oxygen extractors, two radiating heat tables, one suctioning machine, two weighing scales, a pulse oximeter, a glucometer, one refrigerator and a gas cooker. The BRH lacks a neonatal intensive care unit. The neonatology unit is taken care of by a paediatrician, two general practitioners, and eight nurses. Nurse to patient ratio varies from 1:12 in the morning shift to 1:25 in the night shift.

### Participants and sampling

We consecutively enrolled all newborns admitted to the neonatology unit during the study period. Newborns whose parents or guardians denied consent were excluded. On admission, we interviewed their parents or guardians, and then examined these newborns. We monitored their outcome till 28 days after post-menstrual term. When a neonate was discharged prior to 28 days after post-menstrual term, weekly home visits and phone calls were used to monitor the newborn’s progress at home. The minimal sample size was calculated assuming a 20.3% [9] hospital neonatal mortality rate and a precision of 5% [15], hence a minimum of 249 neonates required as study participants.

### Data collection, variables and follow-up

A pre-tested case record form was used to study the following variables;

- **Neonatal characteristics**: age on admission, gestational age (calculated from last menstrual period), Apgar score at 5th minute, presenting complaint, gender, birth weight, and the findings on physical examination (temperature, heart rate, respiratory rate, neurological state, signs of respiratory distress, abnormal breath sounds, heart murmurs, abdominal distension, enlarged abdominal organs, abdominal masses).
- **Maternal characteristics:** maternal age, marital status, employment status, place of residence and occupation.
- **Obstetrical data:** parity, number of antenatal care visits done, prophylaxis during pregnancy, type of pregnancies (multiple or singleton gestation), diseases during pregnancy, mode of delivery (normal vaginal delivery, caesarean section, instrumental delivery), place of delivery, prolonged rupture of membranes, maternal fever, meconium stained amniotic fluid and premature rupture of membranes.
- **Neonatal outcome:** duration of hospitalisation, mode of discharge (discharge alive, referred, discharge against medical advice) and death.

### Definition of terms

Neonatal infections were diagnosed based on the presence of infectious risk factors (prolonged rupture of membranes, maternal fever around the delivery period, maternal urogenital infections during the last month of pregnancy, chorioamnionitis, twin with sepsis), clinical signs of infection (fever, hypothermia, pallor, jaundice, refusal to feed, vomiting, abdominal distension, hypotonia, altered consciousness, convulsions, coma) and any of the following biological criteria: leucocytosis >25,000/mm^3^, leucopenia <5000/mm^3^, platelets <100,000/mm^3^, C-reactive proteins >20 mg/l [16]. Prematurity was defined based on WHO case definition of a gestational age less than 37 completed weeks [1]. Hypoxic ischaemic encephalopathy (HIE) was diagnosed based on the Modified Sarnat-Sarnat Score [17] and an Apgar score ≤ 3 at the 5th minute of life associated with neurological signs: hypotonia, coma or convulsions [18]. Bacterial culture and pH analysis were not used for the diagnosis of neonatal infections and HIE respectively, due to non-availability of the required laboratory apparatus in our health facility at the time of this study. The neonatal hospital mortality rate was defined as the number of deaths occurring among admitted newborns over a given period expressed as a percentage [19].

### Data management and statistical analysis

Data was analysed using SPSS version 20. The threshold of statistical significance set at 0.05. A bivariate analysis using the Chi-square test or Fisher exact test, where appropriate, was performed to determine predictors of neonatal hospital mortality pertaining to neonatal characteristics (gestational age, birth weight, gender, APGAR score, age on admission, place of delivery, respiratory distress, apnoea, hypothermia, anaemia, hypoglycaemia), maternal characteristics (maternal age, level of education, marital status, employment status, place of residence) and obstetrical characteristics (parity, number of antenatal care visits, hypertensive disorders, HIV/AIDS infection, intrapartum fever, duration of rupture of membranes, nature of amniotic fluid, mode of delivery). Variables with frequencies less than 10 were excluded from statistical analysis. Statistically significant predictors of neonatal hospital mortality were then computed in multiple logistic regression to eliminate confounders. Adjusted odds ratios (aOR) and their corresponding 95% confidence intervals (95% CI) were reported. Patients lost to follow-up were excluded from the final analysis.

### Ethical considerations

Ethical authorisations were obtained from the Institutional review Board of the University of Douala, the Regional Delegation of Public Health for the North-West region and from the administrative staff of the Bamenda Regional Hospital prior to the start of the study.

## Results

Out of 337 admissions into the neonatology unit, 332 were enrolled into our study (98.5% response rate). The five neonates not included in the study were due to refusal of consent by their parents. There was no case lost to follow-up.

### Baseline characteristics of neonates

Among the 332 neonates studied, 53% were males, 50.9% were delivered at the BRH and 53.6% were admitted to the neonatology unit of BRH within the first 24 h of life. The mean gestational age was 36 ± 3.9 weeks (range: 25 to 43 weeks). Their average birth weight was 2677.2 ± 923 g (range: Between 600 and 4900 g). Neonates with normal birth weight stood at 53.9%. The mean maternal age was 26.4 ± 5.5 years. The majority of mothers were unemployed (84.9%), married (64.8%) and had at least secondary education (57.8%). The rest of the general characteristics are reported in Table 1.

**Table 1:** Baseline characteristics of the newborns and their mothers

Variables	Number of Newborns (*N* = 332)	Frequency (%)
Gender		
Male	176	53
Female	156	47
Gestational age (weeks)		
Preterm (<37)	111	33.4
Term (37–41)	188	56.6
Post term (≥42)	33	9.9
Age on admission		
0–24 h	178	53.6
1–7 days	103	31
8–28 days	51	15.5
Birth weight		
Low birth weight (<2500 g)	132	39.8
Normal birth weight (2500-3999 g)	179	53.9
Macrosomia (≥4000 g)	21	6.3
Maternal age (years)		
< 20	32	9.6
20–34	266	80.1
≥ 35	34	10.2
Marital status		
Single	117	35.2
Married	215	64.8
Educational level		
No formal education	23	6.9
Primary	124	37.4
Secondary	61	18.4
Higher	124	37.4
Employment status		
Employed	50	15.1
Unemployed	282	84.9

### Causes of neonatal admissions

The leading causes of neonatal admissions were complications of preterm birth (32.2%), neonatal infections (31.3%) and hypoxic ischaemic encephalopathy (14.5%) as illustrated in Table 2.

**Table 2:** Causes of neonatal admissions

Diagnoses	Number of newborns *N* = 332	Frequency (%)
Complications of preterm birth^a^	107	32.2
Low birth weight	66	19.8
Hypothermia	43	13
Infections	27	8.1
Respiratory distress	19	5.7
Hypoglycaemia	16	4.8
Birth asphyxia	10	3
Jaundice	08	2.4
Congenital heart disease	02	0.6
Necrotizing enterocolitis	02	0.6
Neonatal infections^b^	104	31.3
Birth asphyxia^b^	48	14.5
Neonatal jaundice^c^	25	7.5
Intra-uterine growth restriction	21	6.3
Neonatal respiratory distress	08	2.4
Transient tachypnoea of the newborn	06	1.8
Meconium aspiration syndrome	02	0.6
Congenital anomalies	08	2.4
Congenital heart disease^b^	05	1.5
Gastroschisis	02	0.6
Congenital diaphragmatic hernia	01	0.3
Clinical monitoring	07	2.1
Feeding problems^c^	04	1.2
Irritability^d^	01	0.3
Meconium stained amniotic fluid^d^	01	0.3
Birth injury	01	0.3
Neonatal seizures^d^	02	0.6
Hypoglycaemia^b^	02	0.6

^a^69 preterm newborns admitted for complications of preterm birth presented with more than one complication

^b^Excluding cases related to complications of preterm births

^c^Excluding cases related to related to complications of preterm births and neonatal infections

^d^Excluding cases related to neonatal infections and birth asphyxia

### Outcomes of neonatal admissions

**Fig. 1:**
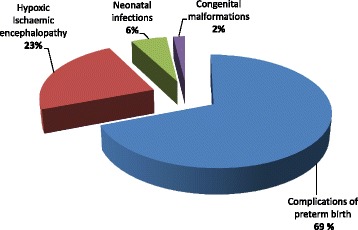
Causes of neonatal hospital mortality (*n* = 52 neonatal deaths)

Two hundred and forty-seven newborns (74.3%) were discharged alive; 52 (15.7%) newborns died in the course of hospitalization; 30 (9%) were discharged against medical advice (DAMA) and three (1%) were referred to a tertiary centre in urban Cameroon for the management of surgical emergencies (two cases of gastroschises and one case of congenital diaphragmatic hernia). The mean duration of hospitalisation in deceased newborns was 2.9 ± 5.4 days compared to 15.1 ± 13 days in those discharged alive (*p* < 0.01). Out of the 30 newborns DAMA, two died at home from complications of preterm birth (mainly hypothermia) and one died at home from deteriorating sepsis, hence, an overall fatality rate of 16.6%. The causes of neonatal hospital mortality were dominated by complications of prematurity (69%), HIE (23%), and neonatal infections (6%) as shown in Fig. 1. In-hospital deaths from complications of preterm birth (69%) were as follows; neonatal infections (34%), respiratory distress (19%), HIE (8%), congenital heart diseases (4%) and necrotising enterocolitis (4%).

### Predictors of neonatal hospital mortality

Using bivariate analysis, factors associated with neonatal hospital mortality were low birth weight (*p* < 0.01), prematurity (*p* < 0.01), Apgar score < 7at the 5^th^minute (*p* < 0.01), attending less than four antenatal care visits (*p* < 0.01), residing in a rural area (*p* < 0.01), age on admission <7 days (*p* < 0.05), and maternal illnesses (*p* < 0.05) as depicted in Table 3. After multiple logistic regression, an Apgar score less than seven at the 5th minute was retained as the lone predictor of neonatal hospital mortality (Table 4).

**Table 3:** Predictors of neonatal hospital mortality at a bivariate analysis

Variable	Dead newborns (*n* = 52)	Newborns discharged alive (*n* = 280)	Odds ratio	95% confidence interval	*p*-value
Neonatal characteristics					
Gestational age					
< 37 weeks	34 (65%)	77 (28%)	4.98	2.66–9.34	<0.01
≥ 37 weeks	18 (35%)	203 (72%)			
Birth weight (grams)					
< 2500	37 (71%)	95 (34%)	4.80	2.51–9.19	<0.01
≥ 2500	15 (29%)	185 (66%)			
Gender					
Male	30 (58%)	146 (52%)	1.25	0.69–2.28	0.4621
Female	22 (42%)	134 (48%)			
APGAR at 5th minute					
< 7	44 (85%)	64 (23%)	18.56	8.31–41.45	<0.01
≥ 7	8 (15%)	216 (77%)			
Age on admission (days)					
0–7	49 (94%)	232 (83%)	3.38	1.01–11.29	<0.05
7–28	3 (6%)	48 (17%)			
Place of birth					
BRH	21 (40%)	148 (53%)	0.60	0.33–1.10	0.1007
Others	31 (60%)	132 (47%)			
Maternal characteristics					
Maternal age (years)					
< 20 and ≥35	13 (25%)	53 (19%)	1.43	0.71–2.86	0.3155
20–34	39 (75%)	227 (81%)			
Level of education					
Primary or less	27 (52%)	120 (43%)	1.44	0.79–2.61	0.2283
Secondary or more	25 (48%)	160 (57%)			
Marital status					
Single	23 (44%)	94 (34%)	1.57	0.86–2.86	0.1415
Married	29 (56%)	186 (66%)			
Occupational status					
Unemployed^a^	44 (85%)	238 (85%)	0.97	0.43–2.21	0.9432
Employed	8 (15%)	42 (15%)			
Place of residence					
Rural area	20 (38%)	47 (17%)	3.09	1.63–5.88	<0.01
Sub-urban area	32 (62%)	233 (83%)			
Obstetrical characteristics					
Parity					
Primiparity and Grand Multiparity	33 (63%)	137 (49%)	1.81	0.98–3.34	0.0564
Multiparity	19 (37%)	143 (51%)			
Number of antenatal visits					
0–3	30 (58%)	86 (31%)	3.08	1.68–5.64	<0.01
≥ 4	22 (42%)	194 (69%)			
Maternal illnesses during pregnancy					
Yes	22 (42%)	73 (26%)	2.08	1.13–3.83	<0.05
No	30 (58%)	207 (74%)			
Maternal fever during labour					
Yes	1 (2%)	16 (6%)	0.32	0.04–2.49	0.2788
No	51 (98%)	264 (94%)			
Duration of rupture of membranes(hours)					
≥ 12	18 (35%)	64 (23%)	1.79	0.95–3.37	0.0736
< 12	34 (65%)	216 (77%)			
Meconium stained liquor					
Yes	4 (8%)	12 (4%)	1.86	0.58–6.01	0.2991
No	48 (92%)	268 (96%)			
Mode of delivery					
Vaginal	46 (89%)	221 (79%)	2.05	0.83–5.02	0.1179
Caesarean section	6 (11%)	59 (21%)			

*BRH* Bamenda Regional Hospital

^a^Housewives, students

**Table 4:** Predictors of neonatal hospital mortality at logistic regression

Variable	Adjusted odds ratio	95% confidence interval	*p*-value
Age on admission 0–7 days	1.77	0.31–10.25	0.527
Birth weight < 2500 g	7.41	0.48–115.31	0.152
Apgar score < 7 at 5th minute	16.41	6.35–42.47	<0.01
Gestationalage < 37 weeks	0.73	0.32–1.68	0.458
Number of antenatal visits <4	1.34	0.51–3.54	0.557
Maternal illnesses during pregnancy	1.66	0.71–3.89	0.248
Residence in rural area	0.55	0.23–1.31	0.180

## Discussion

This study aimed at determining the neonatal hospital mortality rate and its predictors in a major referral hospital in the North-West region, Cameroon. The three main causes of neonatal admissions were complications of preterm birth, infections and birth asphyxia. The neonatal hospital mortality rate was 15.7% and mainly related to complications of preterm birth (69%), birth asphyxia (23%) and infections (6%). A five-minute Apgar score less than seven was an independent predictor of neonatal hospital mortality and increased the odds of neonatal hospital mortality by 16.

In 9% of cases, the parents or guardians of the neonates systematically requested a DAMA mainly because they could no longer afford the cost of neonatal care. With a daily cost of neonatal care estimated at 10–17 U.S. dollars in our setting, this fee was relatively expensive for an average Cameroonian family, given that 9.6% of the population live below the international poverty line [20]. Other points in relation to poverty are that 84.9% of mothers were unemployed and there is no prevailing national health insurance policy for parents who cannot afford health care for their neonates.

The high neonatal hospital mortality rate of 15.7% could be explained by the referrals of more debilitating cases. Furthermore, among the 52 in-hospital deaths, 43 (83.3%) occurred during the first week of life which is very critical in the life of a neonate and warrants close follow-up. Our neonatal hospital mortality rate (15.7%) is superior to the 9.8–10% reported from urban Cameroon [10, 14, 21], and this can be explained by the inequality of neonatal care between the sub-urban and urban health settings of the country.

Discharged neonates were monitored at home up to 28 days after the post-menstrual term. One neonate DAMA died after 5 days from worsening sepsis while two preterm neonates died from hypothermia after 2 days. As a result, the overall mortality rate shifted from 15.7 to16.6%. This finding further re-iterates the need for a national health insurance policy, considering the fact that all neonatal deaths from DAMA were compounded by financial constraints which hindered adequate neonatal care.

The causes of neonatal hospital mortality were dominated by complications of preterm birth (69%), corroborating with contemporary global, regional and national reports [4]. It is projected that complications of preterm birth will probably remain the leading causes of neonatal mortality as well as under-five mortality in 2030 unless full considerations are given to its management [4, 6]. The preponderance of infections, respiratory distress and birth asphyxia among causes of hospital mortality due to complications of preterm birth in our cohort may be explained by inefficacious antibiotic regimens and possible antibiotic resistance. Other reasons could include suboptimal use of antenatal steroids for preterm labour, lack of specific therapy (exogenous surfactant) for respiratory distress syndrome, the insufficiency of oxygen cylinders and oxygen extractors for supportive treatment of respiratory distress, unskilled birth attendants and defective neonatal resuscitation practices for preterm neonates with birth asphyxia. Meanwhile, no fatal outcome was observed in complications of preterm births like hypothermia and hypoglycaemia. This may imply satisfactory means of thermoregulation by incubators and kangaroo mother care for preterm newborns with hyporthermia, effective glucose therapy and breastfeeding practices to control hypoglycaemia in this setting. In contrast, studies from urban Cameroon [9, 21–23] observed a higher proportion of neonatal infection-related deaths (37.5–60%) compared to the of 6% recorded in our study. This could be explained by the recent refresher courses on the hygienic care of childbirth and the postnatal period undertaken by the obstetrical and neonatal staff of our hospital.

An Apgar score less than seven at the 5th minute was an independent predictor of neonatal hospital mortality, concurring with Ekure et al. in Nigeria [24] and Chiabi et al. in urban Cameroon [21]. Noteworthy, this score is often criticized for being subjective [25, 26] and prone to errors in the assessment of neonatal adaptation of preterm neonates, in whom the muscle tone, colour and reflexes partially depend on the physiological maturity of the newborn [27]. However, until a simpler and more useful clinical scoring system for assessing neonates is developed, our finding suggests that an Apgar score less than seven at the 5th minute is associated with inefficient neonatal resuscitation and therefore neonatal mortality.

The main limitation of our study is that it is a single-centre study, though, the findings obtained can still be generalised because they portray similar aetiologies of neonatal hospital mortality observed in urban settings of Cameroon [9, 10, 21] and abroad [28, 29]. The merits of this study include its prospective cohort design and robust statistical methods to provide a contribution of level II scientific evidence [30] to the scarcity of data on neonatal hospital mortality in a sub-urban tropical setting. Other similar studies [14, 23, 31, 32] have been conducted in urban Cameroon using a retrospective design, thus there is a risk that some of the records studied were not correctly filled with resultant inaccurate results on neonatal hospital mortality.

## Conclusion

This is one of the first studies investigating neonatal hospital mortality in a major referral hospital in a sub-urban Cameroon. Its findings suggest that neonatal hospital mortality is still high and accounted by three main pathologies; complications of preterm births, birth asphyxia and neonatal sepsis. Substantial efforts to improve antenatal care, antenatal steroids for preterm labour, sound feeding practices, and early identification and treatment of preterm birth complications could help reduce the burden of neonatal hospital mortality in preterm neonates. Skilled birth attendants and improved neonatal resuscitation schemes may curb the incidence and mortality related to birth asphyxia. Meanwhile, timely detection and treatment of maternal infections, and the use of hygienic childbirth practices may be invaluable in tackling neonatal infections. Also, there should be a reinforcement of refresher courses for health care providers on the management of neonatal infections, birth asphyxia and complications of preterm births. Lastly, a national health insurance policy could be instituted in order to render health care accessible to this vulnerable population. Cost-benefits studies are warranted for this regards. Overall, these policies will go a long way to build sustainable health care systems that could help reduce the neonatal hospital mortality rate in sub-urban Cameroon by 2035.

## Abbreviations

BRH: Bamenda Regional Hospital
DAMA: Discharged Against Medical Advice
HIE: Hypoxic Ischaemic Encephalopathy
NMR: Neonatal Mortality Rate
WHO: World Health Organisation
